# Comparison of characteristics and management of emergency department presentations between patients with met and unmet palliative care needs

**DOI:** 10.1371/journal.pone.0257501

**Published:** 2021-09-27

**Authors:** Scott W. Kirkland, Miriam Garrido Clua, Maureen Kruhlak, Cristina Villa-Roel, Stephanie Couperthwaite, Esther H. Yang, Adam Elwi, Barbara O’Neill, Shelley Duggan, Amanda Brisebois, Brian H. Rowe

**Affiliations:** 1 Department of Emergency Medicine, University of Alberta, Edmonton Alberta, Canada; 2 St. Joseph’s Healthcare Hamilton; Hamilton, Ontario, Canada; 3 Alberta Health Services, Edmonton, Alberta, Canada; 4 Grey Nun Hospital, Covenant Health, Edmonton, Alberta, Canada; 5 Department of Medicine, University of Alberta, Edmonton, Alberta, Canada; 6 School of Public Health, University of Alberta, Edmonton, Alberta, Canada; University of Technology Sydney, AUSTRALIA

## Abstract

**Introduction:**

This study examined emergency department (ED) presentations of patients with end of life (EOL) conditions and patients having met and unmet palliative care needs were compared.

**Methods:**

Presentations for EOL conditions were prospectively identified and screened for palliative care needs. Descriptive data were reported as proportions, means or medians. Bi-variable analysis for dichotomous and continuous variables were performed by chi-squared and T-tests (p≤0.01), respectively. A multivariable logistic regression model identified factors associated with having unmet palliative needs and reported adjusted odds ratios (aOR) with 95% confidence intervals (CI).

**Results:**

Overall, 663 presentations for EOL conditions were identified; 518 (78%) involved patients with unmet palliative care needs. Presentations by patients with unmet palliative needs were more likely to involve consultations (80% vs. 67%, p = 0.001) and result in hospitalization (69% vs. 51%, p<0.001) compared to patients whose palliative needs were met. Patients with unmet palliative care needs were more likely to have previous ED visits (73% unmet vs. 48% met; p<0.001). While medication, procedures, investigations and imaging ordering were high across all patients with EOL conditions, there were no significant differences between the groups. Consultations with palliative specialists in the ED (6% unmet vs. 1% met) and following discharge (29% unmet vs. 18% met) were similarly uncommon. Patients having two or more EOL conditions (aOR = 2.41; 95% CI: 1.16, 5.00), requiring hospitalization (aOR = 1.93; 95% CI: 1.30, 2.87), and dying during the ED visit (aOR = 2.15; 95% CI: 1.02, 4.53) were strongly associated with having unmet palliative care needs.

**Conclusions:**

Most ED presentations for EOL conditions were made by patients with unmet palliative care needs, who were significantly more likely to require consultation, hospitalization, and to die. Referrals to palliative care services during and after the ED visit were infrequent, indicating important opportunities to promote these services.

## Introduction

With an increase in the number of patients presenting to the emergency department (ED) with end of life (EOL) conditions [1–3], research has focused on ways of improving care for these patients. While EDs are capable of treating and managing acute symptoms experienced by patients with EOL conditions, many of these patients would likely benefit from referral to services for their long-term physical, spiritual, psychological, and social care needs [4]. As such, numerous studies have assessed the ability of screening tools to help ED healthcare providers identify ED patients with unmet palliative care needs who may benefit from timely referral to palliative services [4]. The proportion of patients identified as having unmet palliative care needs across various EDs have ranged considerably, from 5% [5] to 83% [6], which is likely due to differences in the criteria for palliative needs among the available screening tools, clinical heterogeneity among the study populations, and variability in healthcare systems. While various studies have identified ED patients with unmet palliative care needs, only a handful of studies have conducted an assessment of whether there are any potential differences in the characteristics and ED management of patients identified as having met or unmet palliative care needs [6, 7]. There is some evidence that patients with EOL or palliative conditions have an increased risk of hospitalization [6, 7] and increased laboratory testing [6]. There is, however, a lack of understanding as to the utilization of available palliative care services [6, 7], as well as the frequency of formal goals of care (GOC), which can impact the care delivered in the ED or in their subsequent hospitalization. Gaining a better understanding of ED patients with unmet palliative care needs may assist emergency physicians, ED staff and health administrators identifying potential gaps in care and ways to improve the services and overall quality of care for patients with EOL conditions [4, 8].

The objective of this study was to compare the characteristics and ED management of patients identified has having unmet palliative care needs (“unmet palliative needs”) using a modified screening tool [9] to those patients identified as not having palliative care needs (“met palliative needs”).

## Materials and methods

### Study design and setting

This prospective cohort study was conducted between March–August 2018 in one major academic tertiary care hospital (University of Alberta Hospital) and one community hospital (Grey Nuns Community Hospital) located in Edmonton, a city with a population of approximately 1 million people in Alberta, Canada. Both ED sites are staffed by full-time emergency physicians with Royal College of Physicians and Surgeons emergency medicine fellowship training or College of Family Physicians of Canada emergency medicine certification. While the University of Alberta Hospital includes a separate adult and pediatric ED, the study was only conducted in the adult ED. The Grey Nuns Community Hospital includes a single ED that provides care to pediatric and adult patients and has a 20-bed palliative care in-patient unit and palliative care coverage is provided by a city-wide service.

### Study participants

Emergency physicians at both study sites were approached by the study team in-person or via email to provide written consent to participate in the study. Between March–August 2018, participating physicians were instructed to screen all ED presentations made by adult patients (17 years and older) during their clinical shifts to identify patients presenting with EOL conditions including cancer, chronic pulmonary disease (COPD), chronic kidney disease (CKD), heart failure (HF), cirrhosis, dementia, and progressive central nervous system (PCNS) disease (e.g., multiple sclerosis, Parkinson’s disease, amyotrophic lateral sclerosis). Clinical judgement was permitted to identify patients with EOL conditions; however, standardized clinical criteria for advanced or end-stage illness for each of the eligible clinical condition were provided to the physicians on the screening form (S1 and S2 Tables) [9, 10]. All presentations for EOL conditions identified by participating physicians during the study period were eligible for inclusion, with the exception of presentations as a result of a hand-over from another participating physician. The hand-over of patients commonly occurs at shift change in over-crowded Canadian urban EDs, and to avoid the risk of screening duplicate presentations, physicians were instructed to avoid screening any patients received in hand-over from another ED physician. Our research staff helped identifying potentially eligible patients using the Emergency Department Information System (EDIS); however, the presence or absence of an EOL condition was confirmed by the patient’s attending physician.

### Screening process

For each presentation for an EOL condition that was identified, participating physicians completed a paper-based screening tool to identify the proportion of presentations made by patients with unmet palliative care needs (S1 Table) [9, 10]. The original screening tool, which had content and face validity [9], was reviewed and modified by a panel of clinical experts (in emergency medicine, intensive care, nephrology, palliative care, and internal medicine) as well as methodologists to gather additional information for the purposes of the current study. To obtain a better understanding of the frequency of presentations to the ED by patients with EOL conditions who could benefit from referral to palliative care services, as well as gain a better understanding of the ED management of patients with and without unmet palliative care needs (e.g., including treatments, disposition, and referrals), presentations for septic shock/severe sepsis or other conditions with a high chance of accelerated death (e.g., intracranial bleed not compatible with life, cardiac arrest, etc.) were removed from the list of eligible EOL conditions on the screening tool. In addition, the screening tool was modified so that physicians could specifically identify presentations for advanced dementia separately from other advanced PCNS conditions (e.g., multiple sclerosis, Parkinson’s disease, amyotrophic lateral sclerosis). The additional questions asked physicians to identify whether patients presented with formal GOC and their opinion as to the appropriateness of each patient’s current GOC based on their presenting condition. Finally, physicians were asked to identify any perceived challenges they faced during the screening process.

During the physicians shifts, physicians screened each patient they attended to for an eligible EOL condition as indicated in step one of the modified screening tool (advanced cancer, COPD, CKF, HF, cirrhosis, dementia, or other PCNS conditions) (S1 Table). Physicians were instructed to indicate one or more EOL condition in step one as applicable. Physicians then completed step two of the modified screening tool which included a list of pre-established risk factors for unmet palliative care needs [9] that included: two or more ED/hospital visits in the past 6 months, uncontrolled symptoms, functional decline, uncertainty about goals of care and/or caregiver distress, and a positive response to the surprise question (SQ) (physician would not be surprised if the patient died within 12 months) (S1 Table). Based on their assessment of the patient, physicians selected the relevant risk factors for each patient. As established with the original screening tool [9, 10], patients with two or more risk factors for palliative care needs were identified as having unmet palliative care needs, while patients with only one or no risk factors identified by physicians were classified as not having unmet palliative care needs (met palliative needs).

### Data collection

Completed data collection forms were retrieved by research assistants. For each patient presentation with a completed data collection form, trained research assistants collected additional data from medical and EDIS records using standardized chart review methods: 1) patients demographics; 2) mode of arrival; 3) acuity at ED presentation [11]; 4) documented GOC for all ED presentations as well as GOC designations (GOC in the ED); 5) documented GOC upon hospitalization among presentations involving admission to hospital (GOC upon admission); 6) procedures (e.g., invasive or non-invasive ventilation, paracentesis, thoracentesis, intubation), investigations (e.g., laboratory and diagnostic tests including blood work, urinalysis, microbiology, imaging, electrocardiogram) and any medications delivered to the patient in the ED (e.g., fluids/electrolytes, oxygen, narcotics, analgesics, antibiotics, corticosteroids) regardless of the route of administration; 7) consultations requested in ED and post hospital referrals, after inpatient admission; 8) ED times (e.g., length of stay [LOS: triage to discharge time] and physician initial assessment [PIA: triage to doctor assessment time]); 9) patient disposition and ED revisits within 30 days of discharge from the ED, or after hospital discharge when the patient was admitted; 10) ED visits within 6 months prior to their enrollment ED visit. To estimate patient acuity at ED presentation, both participating ED sites utilize the five-level Canadian Triage and Acuity Scale (CTAS) system which is applied by experienced triage nurses at presentation and represents acuity as the following: CTAS 1 –resuscitation; CTAS 2 –emergent; CTAS 3 –urgent; CTAS 4 –less urgent; and CTAS 5 –non-urgent [11]. Formal GOC for each ED presentation and hospitalization was documented in the patients charts by the physician or nurse or in a document referred to as a “green sleeve” which documents the patients formal GOC. In Alberta, the various GOC designation orders are documented based on different levels of medical care including R (resuscitative care), M (full medical care with transfer to acute care), and C (comfort care without a goal to prolong life) with patients being able to specify different levels of care within each level. For example, patients designated as an R GOC could specify R1 (patient accepts any appropriate investigations/interventions, resuscitation and admission to the intensive care unit (ICU)), R2 (patient accepts any appropriate investigations/interventions, resuscitation and ICU care ***excluding*** chest compressions), or R3 (patient accepts any appropriate investigations/interventions, resuscitation and ICU care ***excluding*** intubation and chest compressions). Additional details on all of the various GOC designations are provided in S3 Table.

Data extraction for the first 10 medical records were completed in duplicate and reviewed by an experienced clinical research nurse to identify and mediate disagreements and ensure a unified data collection methodology. Basic information on the emergency physicians’ socio-demographic characteristics, training, years of experience and participation rate were self-reported using paper-based survey methods. All study data were entered into REDCap, a secure web platform for managing online surveys and databases (Vanderbilt University, Knoxville, TN, USA).

### Statistical analysis

All analyses were performed using StataCorp, 2015 (Stata Statistical Software: Release 15. College Station, TX). Descriptive data are reported using proportions for dichotomous variables, means with standard deviations (SD), or medians with interquartile range (IQR) for continuous variables, as appropriate. Bivariate comparisons of dichotomous and continuous variables between patients with unmet and met palliative care needs were completed using chi-squared (χ^2^) test and using T-test or Mann-Whitney U test, respectively. Physician responses to the screening tool question regarding their recommendations for appropriate GOC and screening challenges were coded based on themes and frequencies. Due to the multiple tests performed, a statistical significance level was set at *p* < 0.01.

A logistic regression was used to detect trends in relationships between the variables and having unmet palliative care needs and to estimate unadjusted odds ratio (OR) of all variables. Variables with a p < 0.2 were then incorporated in a multivariable logistic regression model and removed from the final model at *p* > 0.05. The variables in the screening tool (e.g., frequent ED visits/hospitalizations, uncontrolled symptoms, functional decline, uncertainty of GOC/caregiver distress, and SQ) were not included in the adjusted model as these factors were used to directly classify patients as having unmet palliative care needs. Unadjusted and adjusted odds ratios (aOR) with 95% confidence intervals (CIs) for each variable were reported.

#### Sample size calculation

The sample size calculation was based on identification of EOL presentations across three study sites; however, the study could not be conducted at one site as planned due to a lack of available research staff. It was estimated that the participating physicians would have to screen 6000 presentations to identify 600–1200 presentations involving patients with EOL conditions. If 200–400 presentations involving EOL conditions were identified per site, the 95% CI of the high (≥ 90%)/low (≤ 10%) estimates of factors in EOL presentations would be +/- 4 to 3% whereas the 95% CI of the moderate (~50%) estimates of factors in EOL presentations would be +/- 7 to 5%, respectively. The total number of adult ED presentations screened by participating emergency physicians during their shifts, in which they were the most responsible provider, was estimated using EDIS, which tracks which emergency physician is providing care to the patients, as well as handovers.

### Ethics

The Health Research Ethics Boards of the University of Alberta and Covenant Health approved the study protocol, study materials, and was granted access to medical records for the conduct of chart reviews without the need for written informed consent from the individual patients (Reference ID: Pro00078882). The Alberta Research Ethics Community Consensus Initiative (ARECCI) screening tool identified this study as having minimal risk. Emergency physicians provided written informed consent to participate in the study and were provided a $50 gift card for participating in the study and reaching a minimum recruitment target of 20 patients.

## Results

### Physician recruitment

The majority of emergency physicians (n = 45/60; 75%) across the two study sites agreed to participate in the study. Most were male (n = 32/45; 71%) and the average age of was 41 years (SD: 9.3). The majority of physicians had a Fellowship from the Royal College of Physicians and Surgeons of Canada (n = 21/45; 47%) or a Certification from the College of Family Physicians of Canada (n = 21/45; 47%). A slight majority (n = 24/45; 53%) of participating physicians had over 10 years of experience in emergency medicine practice. The median number of patients enrolled by the participating physicians was 19 (IQR: 8, 20).

### Patient enrollment and screening

**Fig 1:**
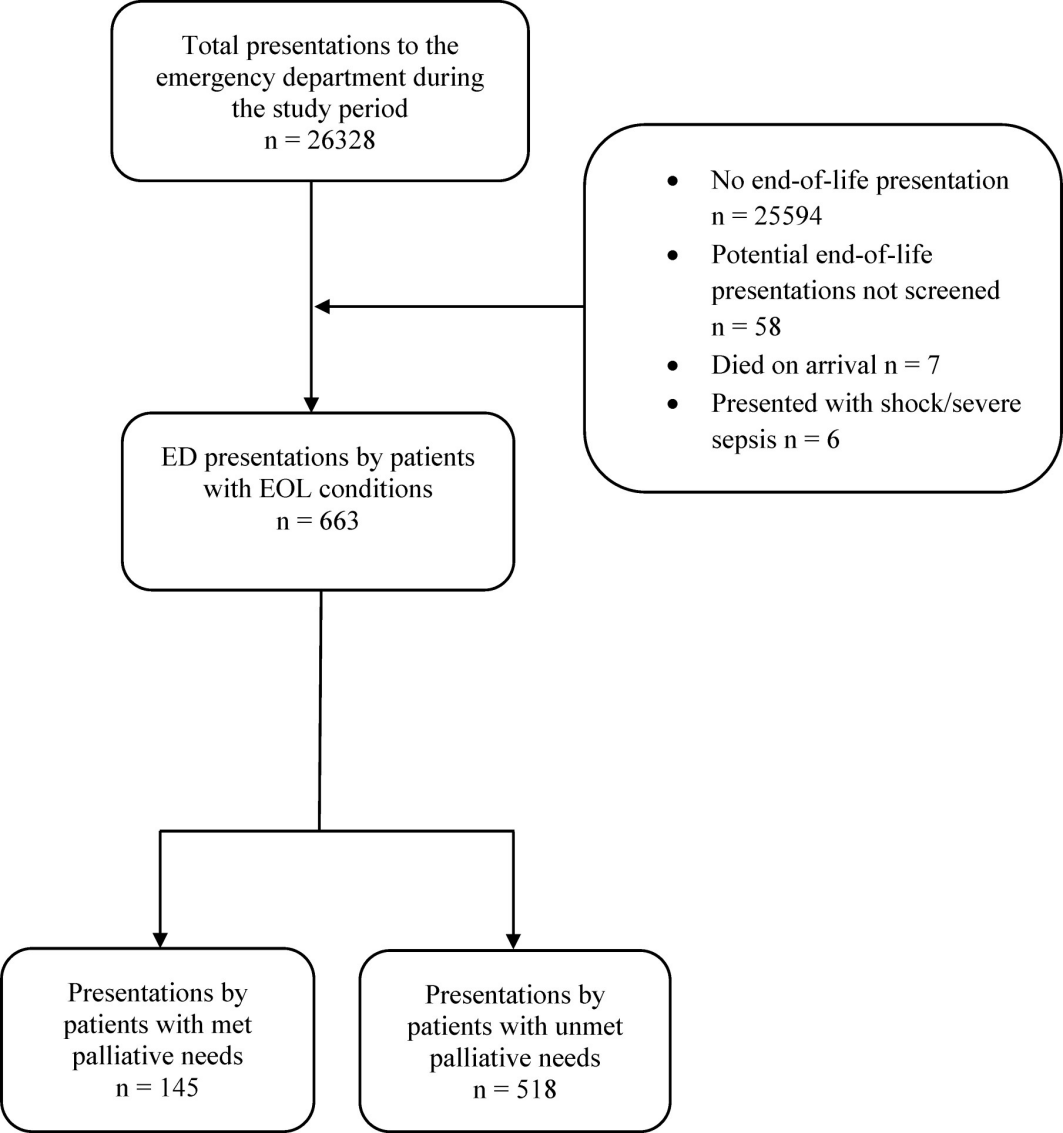
Identification of 663 presentations to the emergency department by patients with end-of-life conditions during the study period.

Over the course of the study period, participating physicians attended a total of 26,328 adult presentations, of which, physicians identified 663 presentations for EOL conditions (see Fig 1). The 663 presentations for EOL conditions consisted of 627 unique patients; the majority of these patients made a single presentation during the study period (95%; n = 594/627). Thirty-three patients presented to the ED multiple times during the study period, with the majority of these patients presenting to the ED twice (91%, n = 30/33). For patients with multiple presentations to the ED, the median number of days between the first and second visits was 30.5 (IQR: 8.5, 46.5). The research staff identified 58 presentations with compatible diagnoses in which the participating physicians did not complete the screening tool (see Fig 1). Across the 663 presentations for EOL needs, 78% of the presentations (n = 518/663) included patients who met the criteria for having unmet palliative care needs (Fig 1).

### Patient demographics

Of the 627 unique patients, 326 (52%) were female and the median age was 76 years (IQR: 63, 85). There were no statistically significant differences between the presentations among patients with met vs. unmet palliative care needs in terms of CTAS scores, mode of arrival by EMS, or presenting to the ED with documented GOC (as documented in the patient medical records) (see Table 1).

**Table 1:** Characteristics and goals of care documentation in a study of 663 presentations to the emergency department by patients with end-of-life conditions, sub-grouped by met and unmet palliative care needs.

Variable	Total presentations (n = 663)	Met needs (n = 145)	Unmet needs (n = 518)	p-value
**Female sex^α^, n/N (%)**	326/627 (52.0)	69/138 (50.0)	257/489 (52.6)	0.596
Age in years, median (IQR)^α^	n = 627	n = 138	n = 489	0.101
	76 (63, 85)	78/138 (64, 87)	75/489 (63, 85)	
**EMS arrival^β^, n (%)**	396/661 (59.9)	86/145 (59.3)	310/516 (60.1)	0.868
**CTAS scores, n (%)**				0.766
**CTAS Score 1**	33 (5.0)	6 (4.1)	27 (5.2)	
**CTAS Score 2**	274 (41.3)	59 (40.7)	215 (41.5)	
**CTAS Score 3**	320 (48.3)	74 (51.0)	246 (47.5)	
**CTAS Score 4**	36 (5.4)	6 (4.1)	30 (5.8)	
**Goals of Care (GOC) documented in patients’ medical records**				
**GOC arrival ED, n/N (%)**	437/661 (66.1)	87/144 (60.4)	350/517 (67.7)	0.103
**R1**	99 (22.7)	24 (27.6)	75 (21.4)	
**R3**	34 (8.0)	8 (9.2)	26 (7.4)	
**M1**	221 (50.8)	36 (41.4)	185 (52.9)	
**C1**	33 (7.6)	9 (10.3)	24 (6.9)	
**Other**	50 (11.4)	10 (11.5)	40 (11.4)	
**GOC upon admission, n/N (%)**	291/424 (68.6)	47/73 (64.4)	244/351 (69.5)	0.283
**R1**	69 (23.7)	18 (38.3)	51 (20.9)	
**R3**	25 (8.6)	4 (4.2)	21 (8.6)	
**M1**	136 (46.7)	17 (36.2)	119 (48.8)	
**C1**	31 (10.7)	3 (6.4)	28 (11.5)	
**Other**	30 (10.3)	5 (10.6)	25 (10.2)	

^α^627 total unique patients after adjusting repeated ED visits

^β^ED Arrival by EMS was not documented in 2 patients; Bolded p-values are considered statistically significant p < 0.05.

Note: CTAS = Canadian Triage and Acuity Scale; ED = Emergency department; GOC = Goals of care; IQR = Interquartile range; SD = Standard deviation.

GOC: R = Resuscitative Care; M = Medical Care, excluding resuscitation; C = Comfort care and interventions, focused on symptom control; R1 = patient is expected to benefit from and accepts any appropriate investigations/interventions that can be offered including ICU care and resuscitation; R3 = patient is expected to benefit from and accepts any appropriate investigations/interventions that can be offered including ICU care, excluding intubation and chest compressions; M1 = goals of care and interventions are for cure or control of illness, excluding the option for ICU care, for non-hospitalized patients, transfer to an acute care facility is considered if required for diagnosis and treatment.; M2 = goals of care and interventions are for cure or control of illness, excluding the option for ICU care, for non-hospitalized patients, transfer to an acute care setting or surgical intervention are not generally undertaken for an acute deterioration but may be considered to better understand or control symptoms; C1 = goals of care and intervention are for maximal symptom control and maintenance of function without cure or control of the underlying condition. Transfer maybe undertaken to better understand or control symptoms. Surgery maybe undertaken to better understand or control symptoms.

### Unmet vs Met palliative care needs

Table 2 summarizes the information collected through the modified screening tool (overall and according to unmet vs. met palliative care needs) for each presentation with EOL conditions. Briefly, a positive response to the SQ was the most common risk factor identified (80% unmet vs. 25% met), followed by uncontrolled symptoms (64% unmet vs. 22% met), and frequent ED visits or hospitalizations (63% unmet vs. 19% met). The majority of presentations for patients with unmet palliative care needs had two (46%) or three (32%) risk factors documented for unmet palliative care needs. Advanced cancer was the most prevalent condition in both groups (42% unmet vs. 37% met). Patients with unmet palliative care needs were significantly more likely to have presented with two or more EOL conditions (15% unmet vs. 6% met, p = 0.007).

**Table 2:** Factors included in a modified screening tool to identify met or unmet palliative care needs among 663 presentations to the emergency department by patients with end-of-life conditions.

Variable	Total presentations (n = 663)	Met needs (n = 145)	Unmet needs (n = 518)	p-value
**Diagnosis, n (%)**				
**Cancer**	272 (41.0)	53 (36.6)	219 (42.3)	0.215
**COPD**	107 (16.1)	19 (13.1)	88 (17.0)	0.261
**CKD**	59 (8.9)	15 (10.3)	44 (8.5)	0.489
**HF**	62 (9.4)	10 (6.9)	52 (10.0)	0.251
**Cirrhosis**	49 (7.4)	4 (2.8)	45 (8.7)	0.016
**Dementia**	151 (22.8)	40 (27.6)	111 (21.4)	0.118
**PCNS**	59 (8.9)	14 (9.7)	45 (8.7)	0.717
**Single EOL condition, n (%)**	578 (87.2)	136 (93.8)	442 (85.3)	**0.007**
**≥2 EOL conditions, n (%)**	85 (12.8)	9 (6.2)	76 (14.7)	**0.007**
**Screening tool risk factors for palliative care needs, n (%)**				
**Frequent ED visits/hospitalizations**	356 (53.7)	28 (19.3)	328 (63.3)	**0.000**
**Uncontrolled symptoms**	362 (54.6)	32 (22.1)	330 (63.7)	**0.000**
**Functional decline**	298 (45.0)	18 (12.4)	280 (54.1)	**0.000**
**Uncertainty of GOC/caregiver distress**	123 (18.6)	1 (0.7)	122 (23.6)	**0.000**
**Surprise question**	452 (68.2)	36 (24.8)	416 (80.3)	**0.000**
**Frequency of GOC, n/N (%)**				
**Does the patient have established GOC?**	396/637 (62.2)	87/139 (62.6)	309/498 (62.1)	0.907
**If yes, are they appropriate?**	235/379 (62.0)	60/82 (73.2)	175/297 (58.9)	0.019
**What would appropriate GOC be? n (%)**	428	85	343	
**M**	212 (49.5)	45 (52.9)	167 (48.7)	0.483
**C**	63 (14.7)	4 (4.7)	59 (17.2)	**0.004**
**R**	31 (7.2)	9 (10.6)	22 (6.4)	0.184
**M or C**	15 (3.5)	2 (2.4)	13 (3.8)	0.519
**M or R**	3 (0.7)	0 (0)	3 (0.9)	N/A
**Were there any challenges during the screening process? n/N (%)**	141/601 (23.5)	31/141 (23.5)	110/141 (23.5)	0.990
**Language barrier**	46 (32.6)	10 (32.3)	36 (32.7)	0.961
**Dementia** *	46 (32.6)	12 (38.7)	34 (30.9)	0.556
**Unable to communicate (intubated, etc.)**	11 (7.8)	4 (12.9)	7 (6.4)	0.230
**Unable to communicate (health issues)**	10 (7.1)	1 (3.2)	9 (8.2)	0.342
**Not documented**	5 (3.5)	1 (3.2)	4 (3.6)	0.913
**Other**	23 (16.3)	3 (9.7)	20 (18.2)	0.258

*Dementia, delirium, confusion, obtunded patient; Bolded p-values are considered statistically significant p < 0.01.

Note: CKD = Chronic kidney disease; COPD = Chronic obstructive pulmonary disease; ED = emergency department; EOL = end-of-life; GOC = Goals of care; HF = Heart failure; N/A = Not applicable; PCNS = Progressive central nervous system.

GOC: M = medical care and interventions, excluding resuscitation; C = medical care and interventions, focused on comfort; M1 = goals of care and interventions are for cure or control of illness, excluding the option for ICU care, for non-hospitalized patients, transfer to an Acute Care facility is considered if required for diagnosis and treatment.; M2 = goals of care and interventions are for cure or control of illness, excluding the option for ICU care, for non-hospitalized patients, transfer to an Acute Care or surgical intervention are not generally undertaken for an acute deterioration but may be considered to better understand or control symptoms; C1 = goals of care and intervention are for maximal symptom control and maintenance of function without cure or control of the underlying condition. Transfer maybe undertaken to better understand or control symptoms. Surgery maybe undertaken to better understand or control symptoms. C2 = goals of care and interventions are for physical, psychological, and spiritual preparation for imminent death with maximal efforts directed at compassionate symptom control. Transfer is generally not undertaken.

Among all presentations, physicians reported that a similar proportion of patients presented with established GOC across both groups (62% unmet vs. 63% met). There was no significant difference in the proportion of presentations in which a physician felt the GOCs were inappropriate considering the patients current condition (66% unmet vs. 59% met). In cases where the physicians were of the opinion that patients current GOC was inappropriate, physicians were significantly more likely to recommend C designations (comfort care) that focused strictly on providing comfort for patients presenting with EOL conditions (17% unmet vs. 5% met, p = 0.004) (see Table 2). Among the 33 patients who made multiple ED presentations during the study period, 45% (n = 15/33) of patients didn’t have active GOC documented in their charts during the initial visit but did have established GOC in their second visit. Finally, the proportion of presentations in which the physician identified significant challenges during the screening process was similar between the groups (24% unmet vs. 24% met). The presence of a language barrier was the most common challenge during the screening process identified by physicians for patients with unmet palliative care needs (34%), while dementia as the most common challenge for patients with met palliative care needs (40%).

### ED management and outcomes

Table 3 summarizes the differences in ED management across the presentations by patients with unmet vs. met palliative care needs. A similar proportion of presentations for EOL conditions with unmet or met palliative care needs received medications, procedures (e.g., paracentesis, thoracentesis, CPAP, intubation) and investigations (e.g., imaging, blood work, urine or microbiology) within the ED (see Table 3). Presentations for patients identified as having unmet palliative care needs were more likely to have an ED-based consultation requested by the attending physician (80% unmet vs. 67% met; p = 0.001); however, the median number of consultations between the groups was similar. There was no difference in the median time from triage to PIA time or the median overall ED LOS between the groups (see Table 3).

**Table 3:** Emergency department management of 663 presentations by patients with end-of-life conditions compared on unmet vs. met palliative care needs.

Variable	Total presentations (n = 663)	Met needs (n = 145)	Unmet needs (n = 518)	p-value
**Medications within the ED**				
**Medications administered^α^, n/N (%)**	559/661 (84.6)	112/143 (78.3)	447/518 (86.3)	0.019
**Number of medications, median (IQR)**	n = 559	n = 112	n = 447	0.422
	3 (2, 5)	3 (2, 4)	3 (2, 5)	
**Most common classes of medication**				
**Antibiotics**	191 (34.2)	41 (36.6)	150 (33.6)	
**Narcotics**	189 (33.8)	31 (27.7)	158 (35.3)	
**Fluids/Electrolytes**	184 (32.9)	38 (33.9)	146 (32.7)	
**Antiemetic**	155 (27.7)	26 (23.2)	129 (28.9)	
**Analgesics**	146 (26.1)	34 (30.4)	112 (25.1)	
**Procedures^α^, n/N (%)**	70/661 (10.6)	11/143 (7.7)	59/518 (11.4)	0.203
**Paracentesis**	21 (30.0)	5 (45.5)	16 (27.1)	
**Thoracentesis**	2 (2.9)	0 (0)	2 (3.4)	
**CPAP**	5 (7.1)	0 (0)	5 (8.5)	
**NIPPV**	30 (42.9)	5 (45.5)	25 (42.4)	
**Intubation**	9 (12.9)	1 (9.1)	8 (13.6)	
**Other**	6 (8.6)	0 (0)	6 (10.2)	
**Investigations, n (%)**	646/663 (97.4)	141/145 (97.2)	505/518 (97.5)	0.867
**Blood work**	624 (96.6)	135 (95.7)	489 (96.8)	
**Urine**	281 (43.5)	65 (46.1)	216 (42.8)	
**Microbiology**	249 (38.5)	41 (29.1)	208 (41.2)	
**Imaging**	595 (92.1)	126 (89.4)	469 (92.9)	
**Other**	3 (0.5)	1 (0.7)	2 (0.4)	
**Imaging, n/N (%)**	595/646 (92.1)	126/141 (89.4)	469/505 (92.9)	0.172
**MRI**	4 (0.7)	0 (0)	4 (0.9)	
**CT scan**	207 (34.8)	40 (31.8)	167 (35.6)	
**X-RAY**	493 (82.9)	100 (79.4)	393 (83.8)	
**US**	58 (9.8)	15 (11.9)	43 (9.2)	
**VQ**	5 (0.8)	1 (0.8)	4 (0.9)	
**ECG**	372 (62.5)	80 (63.5)	292 (62.3)	
**Consultations in the ED**				
**Consult requested, n (%)**	511 (77.1)	97 (66.9)	414 (79.9)	**0.001**
**Number of consults, median (IQR)**	n = 511	n = 97	n = 414	0.305
	1 (1, 2)	1 (1, 2)	1 (1, 2)	
**Total specialty services consulted**				
**Internal Medicine**	170 (33.3)	30 (30.9)	140 (33.8)	
**Family Medicine**	140 (27.4)	24 (24.7)	116 (28.0)	
**Gastroenterology**	45 (8.8)	4 (4.1)	41 (9.9)	
**Cardiology**	40 (7.8)	13 (13.4)	27 (6.5)	
**ICU/CCU**	20 (3.9)	5 (5.2)	15 (3.6)	
**Palliative**	7 (1.4)	1 (1.0)	6 (1.4)	
**ED LOS, hours, median (IQR)**				
**ED LOS**	10.67 (7.45, 16.82)	9.47 (6.78, 15.85)	10.98 (7.6, 17.1)	0.047
**PIA physician assessment**	1.45 (0.65, 3.12)	1.45 (0.53, 3.08)	1.45 (0.67, 3.15)	0.447

^α^ Unable to get the charts of two enrolled presentations

Bolded p-values are considered statistically significant p < 0.01.

Note: CCU = Critical care unit; CPAP = Continuous positive airway pressure therapy; CT = Computerized tomography; ECG = Electrocardiogram; ED = Emergency department; ICU = Intensive care unit; IQR = Interquartile range; LOS = Length of stay; MRI = Magnetic resonance imaging; NIPPV = Non-Invasive positive pressure ventilation; PIA = physician initial assessment; SD = Standard deviation; US = Ultrasound; VQ = Ventilation/perfusion lung scan.

Outcomes regarding differences in patient disposition and post-discharge referral are provided in Table 4. A significantly higher proportion of presentations to the ED by patients with unmet palliative care needs resulted in admission to hospital (69% unmet vs. 51% met; p<0.001). In addition, a significantly higher proportion of patients who died either in the ED or in-hospital during their index visit had unmet palliative care needs (18% unmet vs. 7% met; p = 0.002). A similar proportion of presentations ended with referrals post-discharge between the groups; there were no differences in the targeted specialty.

**Table 4:** Disposition and post-discharge referrals of 663 emergency department presentations by patients with end-of-life conditions compared on unmet vs. met palliative care needs.

Variable	Total presentations (n = 663)	Met needs (n = 145)	Unmet needs (n = 518)	p-value
**Disposition, n/N (%)**				
**Admitted**	425/652 (65.2)	73/144 (50.7)	352/508 (69.3)	**0.000**
**Discharged**	208/652 (31.9)	66/144 (45.9)	142/508 (28.0)	**0.000**
**Transferred**	16/652 (2.5)	5/144 (3.5)	11/508 (2.2)	0.371
**Other**	3/652 (0.5)	0/144 (0.0)	3/508 (0.6)	0.355
**Patient mortality^£^, n/N (%)**	99/627 (15.5)	10/138 (7.3)	89/489 (18.2)	**0.002**
**Post-discharge referral, n/N (%)**	160/550 (29.1)	34/131 (26.0)	126/419 (30.1)	0.365
**Palliative care**	42 (26.3)	6 (17.7)	36 (28.6)	0.199
**Home care**	51 (31.9)	7 (20.6)	44 (34.9)	0.111
**Geriatric assess**	0 (0.0)	0 (0.0)	0 (0.0)	N/A
**Specialist**	48 (30.0)	9 (26.5)	39 (31.0)	0.613
**Rehabilitation**	11 (6.9)	2 (5.9)	9 (7.1)	0.797
**Hospice**	6 (3.8)	3 (8.8)	3 (2.4)	0.079
**Clinic facility**	29 (18.1)	8 (23.5)	21 (16.7)	0.357
**Medical equipment**	6 (3.8)	2 (5.9)	4 (3.2)	0.461
**Other**	4 (2.5)	2 (5.9)	2 (1.6)	0.155
**Patients with a return visit to the ED within 30 days after discharge^α^, n/N (%)**	167/552 (30.3)	38/122 (31.2)	129/430 (30.0)	0.808
**Number of visits (median {IQR})**	1 (1, 2)	1 (1, 2)	1 (1, 2)	0.987
**≥ 2 visits at 30 days (n {%})**	45 (27.0)	10 (26.3)	35 (27.1)	0.921
**Patients with previous ED visits 6 months prior to index visit^α^, n/N (%)**	393/581 (67.6)	60/124 (48.4)	333/457 (72.9)	**0.000**
**Number of visits, median (IQR)**	2 (1, 4)	2 (1, 3)	2 (1, 4)	0.022
**≥ 3 visits at 6 months, n (%)**	167 (42.5)	19 (31.7)	148 (44.4)	0.065
**≥ 5 visits at 6 months, n (%)**	77 (19.6)	9 (15.0)	68 (20.4)	0.291

Note: N/A = Not applicable; ^α £^627 total unique patients after adjusting repeated ED visits

^£^patients who died either in the ED or during their index hospitalization visit

^α^ED visits not applicable because the patient died or lived out of the province. Bolded p-values are considered statistically significant p < 0.05.

Note: ED = emergency department; IQR = interquartile range; SD = Standard deviation.

No differences in the proportion of patients with return visits to the ED within 30 days or the median number of return visits were found between the groups (see Table 4). Interestingly, patients with unmet palliative care needs were significantly more likely to have visited the ED in the six months prior to the index visit (73% unmet vs. 48% met; p<0.001) (see Table 4).

### Adjusted analyses

Variables considered for the logistic regression analysis included: EOL conditions, hospitalization, consultation requested, received medications, mortality, inappropriate GOC, and main diagnosis of cirrhosis. Regardless of their statistical significance in the univariate analysis, some variables (e.g., age, sex) were considered clinically relevant and therefore retained in the final model to correct for possible unidentified age and sex-related confounding (see Table 5). After adjusting for age and sex, having two or more EOL conditions (aOR = 2.41; 95% CI: 1.16, 5.00), requiring hospitalization (aOR = 1.93; 95% CI: 1.30, 2.87), and dying during the index visit (aOR = 2.15; 95% CI: 1.02, 4.53) were strongly associated with having unmet palliative care needs (see Table 5).

**Table 5:** Factors associated with having unmet palliative care needs among 663 presentations to the emergency department by patients with end-of-life conditions.

	Unadjusted OR (95% CI)	P value	Adjusted OR (95% CI)	P value
Age in years	0.99 (0.98, 1.00)	0.215	0.99 (0.98, 1.00)	0.076
Female sex	1.07 (0.74–1.55)	0.718	1.09 (0.74 0 1.58)	0.689
EOL condition				
Single EOL condition	Ref		Ref	
≥2 EOL conditions	2.60 (1.27–5.32)	0.009	2.41 (1.16–5.00)	**0.018**
Hospitalization required	2.19 (1.51–3.20)	0.000	1.93 (1.30–2.87)	**0.001**
Consult requested	1.97 (1.31–2.96)	0.001	-	-
Mortality	2.95 (1.50–5.83)	0.002	2.15 (1.02–4.53)	**0.044**
Medication received	1.74 (1.01–2.79)	0.021	-	-
GOC			-	-
No	Ref			
Yes	0.80 (0.52–1.23)	0.310	-	-
Inappropriate	1.53 (0.88–2.64)	0.131	-	-
Diagnosis of advanced cirrhosis	3.35 (1.19–9.49)	0.023	-	-

Note: CI = confidence interval; EOL = End-of-life; GOC = goals of care; OR = odds ratio; Ref = reference.

Bolded p-values are considered statistically significant p < 0.05.

## Discussion

This prospective cohort study is one of the first studies to identify and compare the ED management of patients identified as having met or unmet palliative care needs. Using a modified screening tool [9, 10], experienced emergency physicians identified 663 ED presentations by patients with EOL conditions during a four-month study period (~3% of the total number of presenters during the study period), of which, a considerable proportion of the presentations were by patients with unmet palliative care needs (n = 518/663; 78%). Over the course of the study period, presentations by patients with unmet palliative care needs were significantly more likely to involve consultations and require hospitalizations, suggesting their ED care seemed to be focused on treating their presenting symptoms and connecting them to specialized services for admission. While no significant differences were identified in other aspects of ED management between presentations by patients with met or unmet palliative care needs, the use of medical treatments, investigations, and imaging was very high within both groups. The most common EOL diagnosis at ED presentation for patients with met and unmet palliative care needs was advanced cancer; however, patients with advanced cirrhosis represent another potential target for timely ED interventions. While the utilization of various procedures, investigations, and diagnostic imaging within the ED was high regardless of whether or not patients had unmet palliative care needs, the proportion of patients referred to consultations with palliative specialists in the ED, as well as post-discharge referrals to palliative services was low among both groups. Finally, patients with unmet palliative care needs were more likely to previously visit the ED and to die either in the ED or in-hospital during the ED visit. The results of our regression analysis identified having more than two EOL conditions and being hospitalized during the index visit as factors strongly associated with having unmet palliative care needs, suggesting that these factors could assist physicians to identify such patients who may benefit from timely referrals to palliative services. Overall, while presentations for EOL conditions require significant ED and hospital resources, presentations by patients with unmet palliative care needs appear to require care in terms of consultations from specialists, hospitalization, and subsequent ED visits.

Importantly, this study identified gaps in regards to the lack of GOC which can impact ED management, as well as the utilization of available palliative services. First, this study is one of the first to assess potential differences in formal GOC between patients with met or unmet palliative care needs. While patients with met or unmet palliative care needs were just as likely to present to the ED with formal GOC, one-third of patients who were admitted still lacked any formal GOC documentation in their charts, suggesting that a substantial proportion of patients facing the risks associated with hospitalization lacked clearly documented advance care planning [12]. Second, the frequency of within-ED palliative consultations, as well as post-discharge referrals to palliative or hospice services, was low regardless of whether or not patients were identified as having unmet palliative care needs. While it is unclear how many patients were already receiving palliative care services, considering the significant proportion of ED presentations by patients with unmet palliative care needs, it appears that more patients could benefit from referrals to palliative services than were receiving them during the study period. Considering that referrals to palliative care are a Choosing Wisely recommendation in US ED’s [13], the results of this study highlights that there is a need for an improved process to identify ED patients with met and unmet palliative care needs and ensuring that each patient is provided with referrals to palliative services, if they are desired.

While numerous studies have applied various screening tools to identify ED patients with unmet palliative care needs [10, 14–20], an in-depth assessment of the potential differences in ED management between the patients identified as having or not having unmet palliative care needs is uncommon. Verhoeff et al. [6] utilized the SQ to identify unmet palliative care needs among ED patients with advanced cancer, and found that patients with unmet needs presented with more symptoms, higher acuity, and died sooner than patients who were not positive for the SQ [6]. Similar to the current study, patients with advanced cancer identified as having unmet palliative care needs were more likely to be hospitalized; however, no differences were identified in terms of ED length of stay or frequency of diagnostic imaging compared to patients with met palliative care needs [6]. In contrast to the current study, however, Verhoef et al. [6] found that patients with unmet palliative care needs were more likely to undergo laboratory testing, which could be the result of differences in the screening tool, as well as the screened patient population compared to the current study. Interestingly, the study found that involvement with palliative care services in the three months prior to the index visit was low between all of the patients with advanced cancer, regardless of whether or not the patient was identified as having unmet palliative care needs [6]. Another study [9] reported that the majority of ED patients with unmet palliative care needs were admitted to hospital, and had no identifiable involvement with a palliative care team [7]. Overall, the current study provided additional support that there are differences in the ED management among patients with EOL conditions, based on their palliative care needs. Additional research is needed to better understand the ED management of patients identified as having unmet palliative care needs to better identify potential gaps in care and strategies to improve not only their ED care, but also potential opportunities for GOC discussions and referrals to in-hospital or community-based palliative services.

### Limitations

There are several important limitations with this study that must be considered. First, multiple efforts were made to reduce the risk of missing patients, including enrolling the majority of available emergency physicians (75%) and providing frequent reminders to screen to participating physicians, however, it appears that it is likely that some patients with EOL conditions who presented to the ED during the study period were missed. Our research staff did identify 58 patients who were treated by participating physicians during the study period who were not screened but had compatible ED diagnoses and there was documentation on EDIS or their medical records of end-stage or palliative condition or active GOC focused on control and comfort care. Second, the clinical and administrative outcome data (e.g., ED times, disposition, return visits) of this study were dependent on the information available in the electronic records and paper charts, and unfortunately, some information such as patient GOC, were inconsistently documented. Third, this study did not employ an independent third party to verify the accuracy of the patients EOL status. While it is possible that some patients may have been misclassified as having an EOL condition, the participating physicians had years of experience in emergency medicine, and as such the risk of differential misclassification of patients is unlikely. Fourth, it is possible that the modifications made to the palliative screening tool could have impacted its diagnostic properties. At this point, however, the diagnostic properties of the original tool, along with other available screening tools to identify ED patients with unmet palliative care needs is not well established [4]. The content and face validity of the original tool, however, has been established by a team of palliative care experts using modified Delphi techniques. While there is some evidence that the SQ alone has moderate sensitivity and specificity to correctly identify patients who will die [6, 17, 20], its ability to accurately identify patients with unmet palliative care needs has not been established. While further validation of the screening tool to identify ED patients with unmet palliative care needs is needed, the risk factors utilized in the current and modified version of the screening tool are likely sufficient to identify patients who could benefit from palliative care services. Given the lack of clarity regarding the accuracy of any one of the available screening tool for identifying ED patients with unmet palliative care needs, further studies are needed to assess and compare the diagnostic properties of the available tools employed for screening patients with unmet palliative care needs in the ED. Lastly, differences in healthcare systems in Canada compared to other countries in the world could limit the generalizability of the results of this study.

### Conclusions

Notwithstanding the above-mentioned limitations, this study found that a large proportion of patients presenting to the ED with EOL conditions have unmet palliative care needs. Overall, while no differences with respect to medications, procedures, investigations, or diagnostic imaging, patients with unmet palliative care needs were significantly more likely to undergo consultations in the ED, require hospitalizations, and die during the index visit. Based on the results of the adjusted analysis, having multiple EOL or palliative conditions or requiring hospitalization were strongly associated with having unmet palliative care needs, which could be used by emergency physicians to identify patients who may benefit from referral to palliative care services. Despite the availability of palliative care services, referrals to a palliative care consultant in the ED or following discharge was low regardless of the status of their palliative care needs (met or unmet). These findings suggest that steps should be taken to ensure that ED patients who could benefit from palliative services are identified and provided these services which can help improve the EOL trajectory.

## Supporting information

S1 Table A copy of the modified palliative care screening tool. (DOCX)

S2 Table Additional modified palliative care screening tool definitions. (DOCX)

S3 Table Goals of care designation orders. (DOCX)
