# Supplementary material for: Comparison of characteristics and management of emergency department presentations between patients with met and unmet palliative care needs
Source: PLoS One. 2021 Sep 27;16(9):e0257501. doi: 10.1371/journal.pone.0257501 (PMC8476017; doi:10.1371/journal.pone.0257501)
Supplement: S1 Table — (DOCX) [file pone.0257501.s001.docx]

**S1 Table. A copy of the modified palliative care screening tool.**

**1.** **Please complete the following palliative care-screening tool for any patient you have identified during your shift as having advanced or end-stage disease. Check all that apply (✔)**

| **Step 1. Diagnosis** | **Potential assessment characteristics** | **Check** |
| --- | --- | --- |
| Cancer | · Metastatic or local incurable;  · Patient is no longer pursuing therapy;  · Patient is told cancer is not curable; or  · Home care or palliative home care are involved in patient’s care. |  |
| Chronic  Pulmonary Disease | · FEV1 <30% (consistent with literature);  · MRC 4 or 5;  · Home oxygen; or  · BIPAP at home. |  |
| Chronic kidney disease | · End stage renal disease on dialysis or refuse dialysis; or  · CKD Stage 4/5 (consistent with literature). |  |
| Heart Failure | · EF <35 (consistent with literature); or  · Class 3 or 4 heart failure. |  |
| Cirrhosis | · Decompensated Cirrhosis (ascites, coagulopathy, encephalopathy, varices, etc.);  ·  MELD >10 (6% mortality 3 months MELD 10-19); or  ·  Hepatocellular carcinoma. |  |
| Dementia | · Requiring significant support with activities of daily living; or  · In long-term care facility. |  |
| Progressive CNS Disease | · Requiring significant support with activities of daily living; or  · In long-term care facility. |  |
| **Step 2. Unmet palliative care needs** | | **Check** |
| **Frequent visits:**  Two or more ED visits or hospital admissions in the past 6 months. | |  |
| **Uncontrolled symptoms (circle):**  Visit prompted by uncontrolled symptom: pain, dyspnea, depression, fatigue. | |  |
| **Functional decline (circle):**  e.g. Loss of mobility, frequent falls, decrease PO, skin breakdown, etc. | |  |
| **Uncertainty about goals-of-care and/or caregiver distress:**  Caregiver cannot meet long-term needs; uncertainty/distress about goals-of-care. | |  |
| **Surprise question:**  You would not be surprised if the patient died within 12 months. | |  |

*Modified from George N, Barret N, McPeake L, et al. Content Validation of a Novel Screening Tool to Identify Emergency Department Patients with Significant Palliative Care Needs. Acad Emerg Med. 2015; 22: 823-37.

**2. Does this patient have established goals of care?**  Yes No

If yes, are they appropriate?   Yes No

If no, what would appropriate goals of care be? ______________________________________

**3. Were there any challenges during the screening process (e.g., patients with mental health conditions, language barriers, etc.)?** Yes No                       If yes, what were they? _______________________

**Form Completed by:** _____________
