# Supplementary material for: Comparison of characteristics and management of emergency department presentations between patients with met and unmet palliative care needs
Source: PLoS One. 2021 Sep 27;16(9):e0257501. doi: 10.1371/journal.pone.0257501 (PMC8476017; doi:10.1371/journal.pone.0257501)
Supplement: S2 Table — (DOCX) [file pone.0257501.s002.docx]

**S2 Table. Additional modified palliative care screening tool definitions.**

| **Step 1. Diagnosis** | **Inclusion Criteria** |  |
| --- | --- | --- |
| Chronic Pulmonary Disease | MRC 4: Stops for breath after walking 100 yards, or after a few minutes on level ground.  MRC 5: Too breathless to leave the house, or breathless when dressing/undressing. |  |
| Chronic Kidney Disease | CKD Stage 4: Severe CKD (GFR = 15-29 mL/min)  CKD Stage 5: End Stage CKD (CFR <15 mL/min) |  |
| Cirrhosis | MELD >10 (6% mortality 3 months MELD 10-19  MELD Calculator: mdcalc.com  (components: Dialysis at least twice in the past week  Creatinine Bilirubin, INR, Sodium) |  |
|  |  |  |
| Heart Failure | Class III: Marked limitation of physical activity. Comfortable at rest. Less than ordinary activity cases fatigue, palpitation, or dyspnea. |  |
|  | Class IV: Unable to carry on any physical activity without discomfort. Symptoms of heart failure at rest. If any physical activity is undertaken, discomfort increases. |  |
