# Supplementary material for: Comparison of characteristics and management of emergency department presentations between patients with met and unmet palliative care needs
Source: PLoS One. 2021 Sep 27;16(9):e0257501. doi: 10.1371/journal.pone.0257501 (PMC8476017; doi:10.1371/journal.pone.0257501)
Supplement: S3 Table — (DOCX) [file pone.0257501.s003.docx]

**S3 Table. Goals of Care Designation Orders.**

| R  Medical Care and interventions, including resuscitation followed by Intensive Care Unit | R1 |  | Patient is expected to benefit from and is accepting any appropriate investigations/interventions that can be offered including the option of ICU care and resuscitation. |
| --- | --- | --- | --- |
|  | **R2** |  | Patient is expected to benefit from and is accepting any appropriate investigations/interventions that can be offered **including the option of ICU care and intubation, but excluding chest compression.** |
|  | **R3** |  | Patient is expected to benefit from and is accepting any appropriate investigations/interventions that can be offered **including the option of ICU care but excluding intubation and chest compression.** |
| M  Medical Care and interventions, excluding resuscitation | **M1** |  | Goals of Care and interventions are for cure or control of illness, **excluding the option of ICU care.**  **For non-hospital patients, transfer to an Acute Care facility is considered if required for diagnosis and treatment.** |
|  | **M2** |  | Goals of Care and interventions are for cure or control of illness, **excluding the option of ICU care.**  **For non-hospital patients, transfer to an Acute Care or surgical intervention, are not generally** undertaken for an acute deterioration **but may be considered in special circumstances to better understand or control symptoms**. |
| C  Medical Care and interventions, focused on conform | **C1** |  | Goals of Care and intervention are for maximal symptom control and maintenance of function without cure or control of underlying condition. **Transfer may be undertaken in order to better understand or control symptoms. Surgery may be undertaken in special circumstances to better understand or control symptoms.** |
|  | **C2** |  | Goals of Care and interventions are for physical, psychological and spiritual preparation for imminent death (usually within hours or days). **Maximal efforts directed at compassionate symptom control. Transfer is usually not undertaken.** |
